# Identifying and mitigating the biological effectors of the social determinants of health in cancer

**DOI:** 10.1093/jnci/djaf141

**Published:** 2025-06-13

**Authors:** Monica Wagner, Christopher L Benson, Samilia Obeng-Gyasi, Suzanne Conzen, Lauren E Mccullough, Chanita Hughes Halbert, Stanton L Gerson

**Affiliations:** Case Western Reserve University Frances Payne Bolton School of Nursing, Cleveland, OH, United States; Case Western Reserve University Comprehensive Cancer Center, Cleveland, OH, United States; Department of Population and Quantitative Health Sciences, Case Western Reserve University School of Medicine, Cleveland, OH, United States; Case Western Reserve University Comprehensive Cancer Center, Cleveland, OH, United States; Department of Surgery, Ohio State University, Columbus, OH, United States; Division of Hematology & Oncology, Simmons Cancer Center, University of Texas—Southwestern at Dallas, Dallas, TX, United States; Department of Epidemiology, Rollins School of Public Health, Emory University, Atlanta, GA, United States; Department of Population and Public Health Sciences, Norris Comprehensive Cancer Center, University of Southern California, Los Angeles, CA, United States; Case Western Reserve University School of Medicine, Cleveland, OH, United States

## Abstract

Recognizing the interconnectedness between social determinants of health (SDOH) and biological factors associated with cancer, the research community is working to identify and refine biological markers of SDOH that can help us better understand this complex interaction. The National Academies of Science, Engineering, and Medicine convened a workshop with the intent of exploring this interaction and how these factors affect cancer onset and cancer-related health outcomes.^1^ Workshop presentations and discussions provided an overview of biological effectors and SDOH; emerging research in these areas, including the development and validation of biomarkers and strategies to improve the evidence base for monitoring, diagnosing, policy, research, and clinical practice opportunities to improve health and address the impact of SDOH on cancer risk, diagnosis, and outcomes.

## Introduction

A simple question with an undoubtedly complex answer is, “How might we connect the *drivers* of social determinants of health linked to cancer and cancer risk to the biological *drivers* that induce the molecular changes that initiate, promote, and influence progression of cancer?” This is not a question of cause and effect but of interrelatedness between our genetics and lifestyle, exposures and social habits, and those socially derived factors that directly influence our biological and physiological response. The effects of carcinogens are not new, as scientists have long been making these connections.^2^ As far back as the 18th century, it was discovered that chimney sweeps were at increased risk for scrotal cancer due to soot exposure, and today we no longer question whether smoking can cause cancer, nor whether smoking cessation reduces risk and improves treatment responses to cancer. Social scientists tend to examine the social condition(s) (ie, violence, poverty, stress, lack of medical care access and appropriate treatments) and link these to cancer risk, whereas biological scientists start with biological pathways (ie, signaling pathways, stress response molecules such as epinephrine and norepinephrine, altered hormone balance, energy metabolism, omics, genetics and epigenomics). In addition, environmental scientists examine how climate and climate change, environmental toxin exposures, particular matter, microplastics, and bioactive materials such as estrogen mimics influence cancer biology. By building a team science approach that combines the efforts from each perspective with epidemiological tools, we can use a multidimensional approach to collectively identify these drivers and craft well-rounded health policies. Combinatorial methods may uncover pathways in this complex system that were undistinguishable using a singular viewpoint. Furthermore, we are able to ask the following questions:

How does poverty act as a carcinogen? How do social and individual stressors alter cancer induction and progression pathways? How do these social factors imprint our epigenome in a way that predisposes an individual to cancer over generations? What social interventions and intermediate markers can we develop to track and effectively mitigate cancer risk? Are there pathways, such as the adrenergic and/or cortisol response to social and environmental stress exposures, that can be modified to reduce cancer risk and increase treatment response?

## Perspective

Answers to these questions could be forthcoming but will require intense study. Poverty, as a component of the social determinants of health (SDOH) can induce biological changes that become causal in disease onset or progression of cancer. Poverty also limits an individual’s ability to achieve a timely diagnosis of cancer due to limited healthcare access and reduced quality, contributing to the mortality rate in cancers that are easily curable with established screening practices. In tandem with biology, social stressors affect cellular, tissue, and whole-body responses that can accelerate the aging process and, as a result, could lead to earlier onset of cancers that were once primarily seen in older populations. Concepts such as allostatic load and weathering—linked to physiological neuroendocrine stress responses—suggest that chronic exposure to adverse SDOH can alter pathophysiology and contribute to disease cancer initiation and progression.^3^ These social causations can also indirectly and directly affect circadian rhythm, energy metabolism, and fat storage; affect the cortisol and adrenergic stress responses; affect generation of reactive oxygen species; alter DNA configuration; and contribute to inducing acquired mutations and epigenetic changes. The cumulative effects of economic and social marginalization can also generate additional social stressors along with unhealthy habits and lifestyles (including poor nutrition and sleep quality) that develop in response to stress.^4^ The collective “dose” of marginalized living environments, social stressors, and deleterious lifestyle habits may manifest as altered genetic and protein biomarkers relevant to carcinogenesis. At the moment, the causal association between these socio-environmental factors and cancer biology remains understudied but could be useful targets for monitoring effectiveness of policy, behavioral, and pharmacologic interventions.

## Forward-looking approaches to address the biological effectors of SDOH

A constructive goal of advanced societies is to promote environments (physical, social, and economic) that optimize health for everyone. A number of schema reflect a focus on social determinants that identify common elements of health stressors such as Healthy People 2030,^5^ the Health Equity Explorer,^6^ and Toxic Stress Schema,^7^ each of which provides a sociologic stress list that can be tracked in populations and linked to biological measurements, to health outcomes, and to physiologic measurements of stress. As such, it is crucial to conduct research that examines the multilevel influences that can be targeted to decrease cancer incidence and improve outcomes and to do so by linking physiologic measures that mediate cancer onset and progression. The biomarkers can be monitored during interventions in clinical trials. A recent review by Woodruff^8^ noted a long list of social factors adversely affecting health, including access to health care, food insecurity affecting nutrition, smoking and alcohol consumption, and stress and violence affecting day-night cycles and sleep. Likewise, proximity to environmental risks from toxins and particulate matter (highway and car exhaust, industry runoff, and air pollutants) and climate (ambient temperature, floods and disruption of water supplies and sewage, humidity and sun exposure) globally affect health and may trigger cancer initiation or progression. Social drivers appear to induce a multiplex of biological effectors promoting earlier cancer initiation and faster disease progression. Recent reviews^2^^,^^5-7^ provide frameworks that include a multilevel approach to improving cancer outcomes that consider the individual’s molecular and physiological changes and the social and political realms to help us understand the relationships between these levels and how this connectedness ultimately affects human health, and often cancer biology. The biological measurements that can be used in multilevel research with interventions at the individual, neighborhood/environmental, health system, and policy levels are lagging.

To understand and address the issues surrounding SDOH and cancer outcomes, it will be essential for transdisciplinary teams to establish a multilevel framework.^5-7^ These teams work to identify biomarkers and early detection and are crucial in cancer research as they enable timely diagnosis, improving treatment outcomes and survival rates. Frameworks linking biological, behavioral, physical, and sociocultural elements to both individual and community factors will identify the complex biological contributions to predisposition and acceleration of cancer etiology and progression.^9^^,^^10^

Examples of the biological processes affected by SDOH, leading to potential “effector” drivers of and risk for cancer, include epigenomic changes resulting from stress, social isolation, and biological signaling molecules—including cytokines, C-reactive protein, episomes, cortisol, epinephrine, norepinephrine, and reactive oxygen species—all contributing to premalignant cell changes.^7^ These cellular and physiological changes, in addition to gene expression changes, need more in-depth discovery research. Collectively, these factors contribute to the “allosteric load,”^11^ which has been defined as physiologic dysregulation secondary to exposure to chronic stress. For example, neighborhood deprivation^9^ has been associated with alterations in DNA methylation in breast tissue and breast cancer models,^12^ suggesting that social isolation and its physiological sequelae disrupt mammary tissue differentiation during puberty and young adulthood, which laboratory models suggest increase the risk and accelerate the course of breast cancer in later life.^13^ Opportunities exist to leverage population-based research and real-world data from electronic health records to increase understanding of these environment-gene expression-cancer associations. A better understanding of observed population regional differences, age and sex differences, demographics, and urban and rural factors will support the creation of rational policies and directed interventions that enhance early detection and treatment, leading to better biological outcomes as well as reducing overall cancer mortality and morbidity across diverse populations.

## Conclusion

The urgency of these issues is clear; we must take a comprehensive, transdisciplinary approach to alleviate the burden of cancer across communities. Collaboration among diverse scientific disciplines is essential. Equipping biologists with insights into social determinants of health fosters a holistic, patient-centered perspective while providing community health experts with a deeper understanding of cancer’s biological drivers will greatly strengthens efforts to implement rationale programs that combat the disease. Individuals living in communities that face adverse SDOH often experience delayed diagnoses, poorer treatment, and lower survival from otherwise curable cancers. Identifying biological factors may offer predictive markers to evaluate during social and medical interventions to track cancer risk and improved outcomes. A transdisciplinary collaboration—integrating biologists, social scientists, policy experts, and community voices—is a forward-looking approach to establish research priorities and interventions that will have measurable benefits.

## Data Availability

As a commentary perspective, no primary unpublished data are herein presented or accessed.
